# From Platform to Knowledge Graph: Evolution of Laboratory Automation

**DOI:** 10.1021/jacsau.1c00438

**Published:** 2022-01-10

**Authors:** Jiaru Bai, Liwei Cao, Sebastian Mosbach, Jethro Akroyd, Alexei A. Lapkin, Markus Kraft

**Affiliations:** † Department of Chemical Engineering and Biotechnology, University of Cambridge, Philippa Fawcett Drive, Cambridge CB3 0AS, United Kingdom; ‡ Cambridge Centre for Advanced Research and Education in Singapore (CARES), CREATE Tower #05-05, 1 Create Way, 138602 Singapore; ¶ School of Chemical and Biomedical Engineering, Nanyang Technological University, 62 Nanyang Drive, 637459 Singapore; § The Alan Turing Institute, London NW1 2DB, United Kingdom

**Keywords:** Knowledge graph, digital twin, chemistry digitalization, closed-loop optimization, laboratory automation

## Abstract

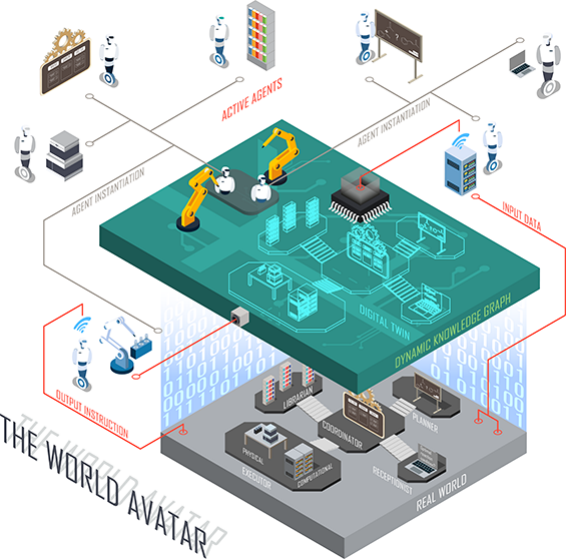

High-fidelity computer-aided experimentation is becoming more accessible with the development of computing power and artificial intelligence tools. The advancement of experimental hardware also empowers researchers to reach a level of accuracy that was not possible in the past. Marching toward the next generation of self-driving laboratories, the orchestration of both resources lies at the focal point of autonomous discovery in chemical science. To achieve such a goal, algorithmically accessible data representations and standardized communication protocols are indispensable. In this perspective, we recategorize the recently introduced approach based on Materials Acceleration Platforms into five functional components and discuss recent case studies that focus on the data representation and exchange scheme between different components. Emerging technologies for interoperable data representation and multi-agent systems are also discussed with their recent applications in chemical automation. We hypothesize that knowledge graph technology, orchestrating semantic web technologies and multi-agent systems, will be the driving force to bring data to knowledge, evolving our way of automating the laboratory.

## Introduction

The automation of the laboratory involves linking the abstract concepts of chemical processes and the hardware responsible for the execution.^1,2^ It can be achieved by creating a fully connected virtual representation of the physical equipment and their status, that is, a “digital twin” of the laboratory that bridges the gap between the virtual and the real world. By doing so, it enables the orchestration of physical and computational experimentation in cyberspace, facilitating the automation of chemical discovery.^3^ Therefore, it shortens the time span from making a new chemical in the research environment to the delivery of its mass production to the end-users. This presents the opportunity to deliver a significant level of decarbonization with reduced labor and energy consumption, making the digitalization of chemical manufacturing one of the critical technology paths toward a more sustainable society.^4,5^

The first automated hardware for chemistry dates back to the late 1960s.^6^ Since then, considerable advances have been made to expand the potentialities of such a tool, covering the fields of chemical reactions,^7,8^ drug discovery,^9^ and material discovery for clean energy.^10,11^ As chemists quest to achieve a universal organic compound synthesis machine, three key capabilities were identified,^12^ that is, access to a database of chemical reaction knowledge, synthetic steps planning, and automated execution of a proposed action sequence. For a detailed historical excursus, the readers may refer to Dimitrov et al.^13^ In 2018, Aspuru-Guzik and Persson^14^ proposed Materials Acceleration Platform (MAP), a platform-based approach, as the paradigm to accelerate the material discovery process, which was further adopted and expanded by Flores-Leonar et al.^15^ In line with the three key capabilities that seem to be required to build a robo-chemist,^12^ Flores-Leonar et al.^15^ envisaged integration of machine learning (ML) algorithms and robotics platforms, with further interfacing between humans and robots, as the way toward autonomous experimentation. The current practices of development toward laboratory automation are seen to be following this trend. Researchers adopt automation of chemical experiments and advances in ML to enable functional material discovery,^16,17^ the discovery of chemical reactions,^18^ synthesis planning,^19,20^ and optimization of process conditions.^21−23^ Despite the great success demonstrated by the community, the effort required to incorporate new equipment into an existing platform can be expensive. Tailored extraction–transformation–loading (ETL) tools and the specific data exchange scheme for establishing effective communication are to be developed for each piece of equipment added. Therefore, these platforms normally face difficulties in scalability and interoperability due to heterogeneous data formats as an obstacle to holistic integration, especially when it comes to the vision of a globally integrated collaboration network.^11^ As a prerequisite condition toward digitalization, the absence of standardized data representation and exchange protocols is seen as one of the critical challenges faced by the community.^8^

A way forward may be offered by Semantic Web technologies,^24^ which present a vision of a fully linked web of data, demonstrating interoperability across scales and domains. It uses ontologies to describe the concepts and relationships within a given domain for communal understanding. In this perspective, we refer to ontologies developed to describe knowledge in the chemistry domain, and more importantly, those implemented in a way that is compatible with the semantic web standards,^25^ as chemical ontologies. One prominent example is ChEBI.^26,27^ An ontology normally consists of two components: a terminological box (TBox) and an assertional box (ABox).^25^ TBox refers to the description at a conceptual level, while ABox stores the data that is a realization of the concepts defined by the TBox. Both levels can be accessed via internationalized resource identifiers (IRIs), essentially generalized uniform resource identifiers (URIs), for unambiguous identification. In the context of automating experiments, this opens up the possibility of developing a fully linked data representation for the chemical processes and equipment status as a universal framework to facilitate concrete data exchange within and between platforms.

Besides the interoperable data representation, an effective way to communicate and share data must be addressed to achieve laboratory automation. In this regard, collective intelligent agents have been used to automate the tasks involved in crystal-structure phase mapping,^28^ material discovery,^29^ and reaction optimization.^30^ Considering the historical discussions of integrating the two technologies,^31^ we hypothesize that an ontological representation of a laboratory, linked with different data standards, would enable the rapid implementation of artificial intelligence (AI) tools for chemical discovery and development.

This perspective aims to review the potential for arising technologies to enhance how we approach laboratory automation. The presentation of this perspective is structured as follows. First, we review the state-of-the-art in laboratory automation practice with a focus on data infrastructure. Based on the limitations of current approaches, we assess community efforts toward standardized data representation and effective data exchange. We identify dynamic knowledge graphs, that is, a combination of ontologies and agents, as an interesting technology option. This approach allows the intelligent automation of experiments to be linked with chemical knowledge resources and aligned with other AI techniques. It is suggested that this will play a key role in the next generation of laboratory automation.

## Platform-Based Approach

**Figure 1:**
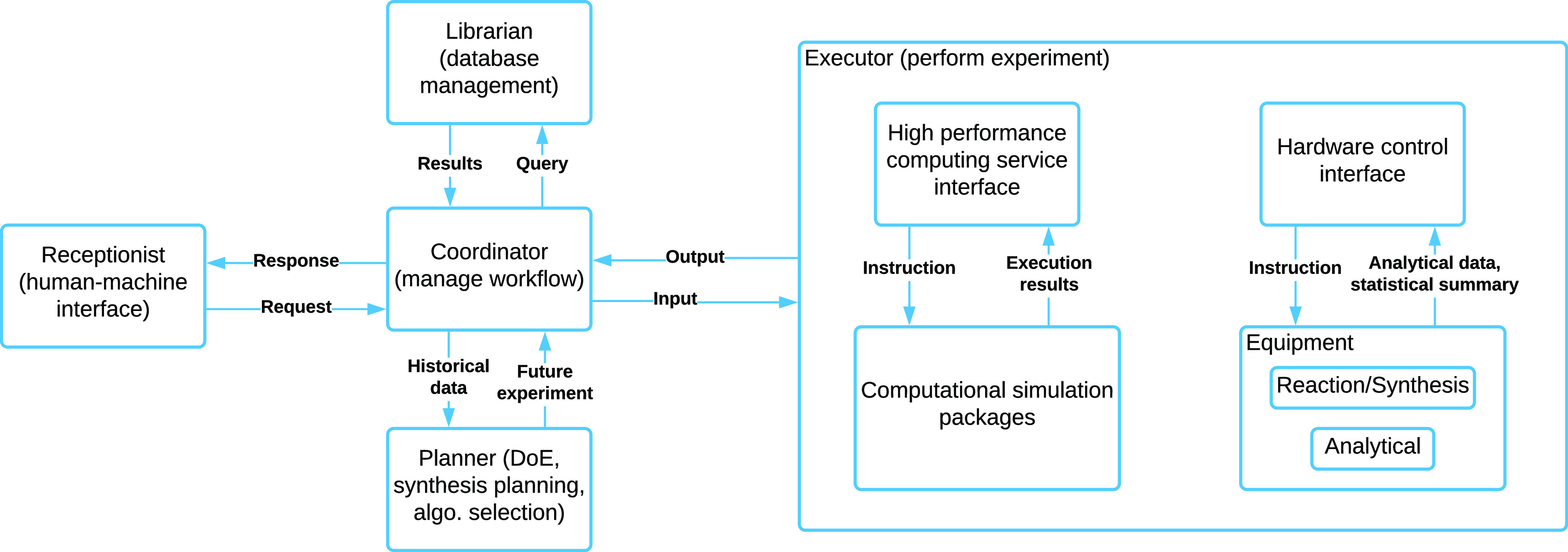
Functional components of a platform-based approach toward chemical discovery, annotated with the communications between each component.

Detailed reviews of the applications of the closed-loop optimization have been published by Cao et al.^32^ and Coley et al.^7^ In this section, we focus on the data flow between the different components of such an automated experimentation platform as presented in the state-of-the-art studies. To have a clearer demonstration of the data flow between different parts, thus revealing how these functional components can be shifted into agents as in the knowledge-graph-based approach, we regroup the five key elements proposed by Flores-Leonar et al.^15^ and recast them as illustrated in Figure 1. The receptionist acts as a human–machine interface that receives, analyzes, and translates the requests into machine-understandable objects, as well as enables real-time and interactive communication between user and data. The coordinator manages the workflow by locating resources given constraints, requesting data from the librarian, asking the planner for suggestions over the next steps, and requesting experiments from the executor. The planner is a decision making entity that designs the experiment, plans retrosynthesis steps, and also selects suitable surrogate models given use-cases. The librarian is responsible for data management, including maintenance of the database, data cleaning, data validation, and outlier detection. The executor performs the computational and physical experiments, both interfaced with the available experimental resources. We categorize the selected studies into the realization of functional components and assess the data communication between each of them. It should be noted that we do not cover the specific internal realization of the components, that is, we do not consider how the planner handles the input historical data and how it recommends the synthesis route; instead, we focus on the format of the recommendation output from the planner. Following the review, we list the limitations of the platform-based approach that lead to the quest for better data representation and exchange protocols.

### Selected Studies

There have been extensive reviews on developing each of the functional components.^15,33−36^ In the context of chemical automation, Mateos et al.^37^ reviewed the realization of the components in selected continuous flow platforms. In this perspective, we selected the studies below to illustrate how the data is exchanged between the functional components in the platform-based approach. Specifically, we will review the data exchange protocols between the coordinator, librarian, planner, and executor for further investigation on interoperability within one platform and between different platforms in the current setups. We identified three main types of data representation and storage in the automated experimentation platforms, namely, variables stored in a reserved memory location of programming languages, data stored in a file on a hard disk, and data stored in a database. Based on this classification, three types of data transfer and communication protocols were identified as assigning in-memory cache values during software program run-time, file transfer protocol, and HTTP request/response. It should be noted that although both of the latter two ways of communication belong to the application layer in the TCP/IP model, they are distinguished herein to emphasize the format in which the data is stored and consequently transferred. To the best of our knowledge, the complete details are summarized in tables in the Supporting Information.

#### Receptionist

The receptionist acts as the human–machine interface. Among different platforms, multiple ways of interaction have been reported. Knight et al.^38^ present a voice-controlled user interface integrating voice, text, and visual dashboards. This increased the flexibility for the experimentalist to communicate and collaborate with the automated setups without coding experience required. Web interfaces via HTTP requests/responses^21,39,40^ is another way of interaction. The advantage of this approach is that authorized users can log in to the web page and access the platform from all over the world.^35^ Moreover, the natural language processing (NLP) modules can build on top of the web interface as chatbots, which can further connect to existing messaging services such as Gmail, Twitter, Slack, and Dropbox.^16,41^ The graphical user interface (GUI) is a more intuitive way of interaction between the users and the automated experimental platforms. It can be built through different coding software, such as Matlab,^42^ Python,^17,19^ and LabVIEW.^22,43,44^ It should be noted that each receptionist can only work within its own operating system due to its bonded communication protocols as well as the coding language.

#### Coordinator

The coordinator manages the workflow in the closed-loop system. Among the different programming languages and tools that have been employed to develop the coordinator, Python is perhaps the most widely adopted. The Aspuru-Guzik group proposed ChemOS,^16,41^ a modular coordinator orchestrating the learning module (the AI-based planner), the communication module (server-based receptionist), and an operation module for remote control of the robotic platform. ChemOS demonstrated decision-making capabilities in managing the workflow for thin-film material discovery^16^ and increasing the efficiency of organic photovoltaics.^45^ It has now been commercialized as Atinary SDLabs^46^ with a Scientia version freely available for academics. Zhu’s group presented MAOSIC,^17^ a coordinator upgraded from their previous system MAOS,^47^ which was applied to the autonomous discovery of optically active chiral inorganic perovskite nanocrystals. Experiment Specification, Capture and Laboratory Automation Technology (ESCALATE) has a coordinator acting as a bridge to connect the experimental workflow.^48^ Its initial implementation was designed for the exploratory synthesis of single-crystal metal-halide perovskites. Further discovery of the formation of two new perovskite phases was demonstrated.^49^ Chemputer^19^ was developed for organic synthesis optimization in batch reactors. This coordinator brought together synthesis abstraction, chemical programming and hardware control, and tested the synthesis of three small pharmaceutical compounds with similar yields to those obtained by manual work. Moreover, by using a standardized format for reporting a chemical synthesis procedure within the coordinator, Chemputer captures synthetic protocols as digital code that can be further published, versioned, and transferred flexibly.

LeyLab^39^ is a PHP-based coordinator orchestrating multiple users and equipment in different continents for the development of catalysts and process conditions in flow reactors. The firewall within the coordinator prevents malicious attacks from unauthorized users.

The Lapkin group presented a Matlab-based coordinator for multi-objective optimization of the reaction conditions for S_N_Ar and N-benzylation reactions.^50^ It demonstrated its flexibility to a different chemical system with an aldol condensation reaction optimization.^42^

There are also coordinators based on LabVIEW. Given the user-friendly graphical programming interface in LabVIEW, building a receptionist module is not required in this setup. However, Matlab^43^ or Python^44^ are occasionally paired up with the LabVIEW to enable the planner module to suggest new experiments.

Another notable development is C#-based ARES OS,^51^ an open-source software released by Air Force Research Laboratory (AFRL) following their autonomous research system (ARES). As the first reported autonomous experimentation system for materials development, ARES demonstrated its capability in carbon nanotube synthesis experiments^52,53^ and additive manufacturing applications.^54^

It can be seen that coordinators followed different coding philosophies in different programming languages. For each case study, the reported coordinator indeed satisfied the specific need yet failed to extend to other systems.

#### Coordinator–Librarian

The interaction between the coordinator and librarian focuses on reading historical data and writing new data for data storage. Depending on the operating system of the coordinator, as well as the structure of the librarian, in each platform, the data communication protocols between the coordinator and librarian are various.

An intuitive approach is to store and transfer the data as variables in the memory of the operating system. Jeraal et al.^42^ stored and transferred data as Matlab variables. Similarly, Christensen et al.^55^ used Python variables for communication. This approach is lightweight and independent of the database structure. However, it is vulnerable as there is no backup for the data obtained. Moreover, the data stored are hard-coded and picked beforehand, meaning the variables will be reassigned during the iterations.

File transfer is an approach to overcome this issue. Cao et al.^5,32^ used CSV files as the bridge for communication. Other studies used MAT files in a similar fashion.^22,56^ In this approach, the experimental results were exported and stored as a file that can be loaded later for suggesting the next experiments. Compared to storing data as in-memory cache variables, the file transfer approach gives a way to back up the data on a separate machine or online server with flexible access and secure storage. However, the files can still be hard to track and classify when the number of experiments is high or more than one type of experiment is run on the platform.

Databases provide a solution to efficiently manage large amounts of experimental data. Li et al.^17^ stored long-term data through SQLAlchemy, which supports a database management system (DBMS), with databases such as MySQL, Postgres, Oracle, and SQLite as the back-end. The coordinator MAOSIC can read and write new entries to the server-based database via API. In Roch et al.,^41^ the coordinator ChemOS was connected to SQLite, and the information was stored in four distinct databases (requestDB, parameterDB, robotDB, feedbackDB) on SQLite to better classify the data and retrieve them in the later stage. Materials Experiment and Analysis Database (MEAD)^57^ consists of both raw data and metadata from high-throughput experimentation. By instantiating an event-sourced architecture for materials provenances (ESAMP),^58^ the MEAD database enabled the ML algorithm to utilize the material state within its experimental workflow for accelerating materials discovery.

#### Coordinator–Planner

To avoid an exhaustive search of the chemical space, the planner needs to decide which new experiments should be conducted. Depending on the purpose of the platform, the planner algorithm can be classified into discovery and optimization. Detailed reviews of the existing algorithms for planner have already been published; interested readers can refer to Garud et al.^59^ and Clayton et al.^60^ The communication between the coordinator and the planner is mainly done in two ways: variables stored in memory^16,22,30^ and file transfer.^5,19,20,50^ It is worth mentioning that the communication protocols are not necessarily the same over one platform. Li et al.^17^ used database queries for the interaction between the coordinator and librarian, yet they depend on Python variables for the communication between the coordinator and planner. It can be seen that the platform-based approach can adapt to different ways of data exchange, yet modifications that are case sensitive will be needed.

#### Coordinator–Executor

The executor runs the experiments, computationally or physically, and sends back the experimental results. The interaction between the coordinator and executor module highly depends on the operating system for the instrument, as the actual experiment resources within the executor are normally surrounded by a layer of interface. Therefore, we review the communication protocols of the physical and computational experimental platforms separately.

##### Physical Experiment Interface

Robotic platforms have their origins in instances such as peptide synthesis^6^ and the pharmaceutical industry.^61,62^ Some existing commercially available semi-automated and fully automated platforms in chemistry have emerged as powerful tools and can be embedded into the closed-loop optimization system.^15^

Commercial platforms provide various high-throughput workflow solutions, ranging from single benchtop or standalone automated workstations up to complete and integrated product development workflows for the entire product development process in chemical material science.^63,64^ Greenaway et al.^65^ applied the Chemspeed Accelerator SLT-100 synthesizer platform in the discovery of porous organic cages and the optimization of the cage formation conditions. This platform can carry out up to 96 reactions in parallel, highly speeding up the testing of the proposed experimental conditions that are sent to the platform via file transfer within the Chemspeed custom software. The hardware from Chemspeed is also used by IBM’s RoboRxn,^66^ a remotely accessible automated organic synthesis platform utilizing various Transformer-based^67^ ML algorithms for chemical reaction prediction,^68^ retrosynthetic pathway planning,^69^ synthesis action extraction,^70^ and chemistry grammar extraction.^71^ Vapourtec delivers an automated flow reaction platform with multiple choices for pumps and flow reactors. Successful examples of using the Vapourtec system in the closed-loop optimization setup include drug discovery,^72^ scale-up development,^73^ and reaction condition optimization.^42,50^ It is worth mentioning that commercially available mobile robots and robotic arms have been used in complex and multistep operations.^20,23^ Communication between the coordinator and the robots was achieved using various communication protocols (TCP/IP over WIFI/LAN, RS-232, websocket, etc.). Although commercial systems developed by various vendors are easily implemented with a user-friendly user interface, it limits the experimental choice across platforms, and it is hard to configure the platform to the existing workflow architecture and setups in the lab.

To enable a modular-based plug-and-play platform, single-board controllers, for example, Raspberry Pi and Arduino, were used to act as the interface layer connecting the coordinator to the actual experiment executor, that is, sample preparation, analytics etc. This is favored by the academic community due to its flexibility and compatibility with different experimental instruments at a relatively low cost. The communication protocols between the coordinator, single-board controller, and experiment executors are various. A TCP/IP protocol was used in the cases where Raspberry Pi was applied. Fitzpatrick et al.^21^ used a VLAN to control lab equipment and also an SSH tunnel between the virtual environment and the remote control server. Similarly, Roch et al.^74^ controlled the pump system using Raspberry Pi and interacted via an SCP with the executor codes. In Chemputer designed by Steiner et al.,^19^ an Arduino was designed as the microcontroller. Instances of experiment executors are created as Python instances at the initialization stage and the coordinator reads related information stored in a GraphML file. Li et al.^17^ conducted their high-throughput experiments via an Arduino control board as well but followed the JSON-RPC 2.0 protocol used for robots and characterization equipment control. A detailed review of microcontrollers and their applications in automated experimental systems can be found in Fitzpatrick et al.^75^ The in-house built platform can connect to different lab equipment based on the users’ need and existing lab setup, yet different communication protocols prevent it from extending to other labs or systems.

Robot Operating System (ROS)^76^ is the *de facto* standard middleware in the robotics field for orchestrating multi-robot systems. In 2019, Marquez-Gamez and Maffetton^77^ proposed a ROS architecture for laboratory robotics motivated by Burger et al.,^23^ envisaging a “cobot” future where human researchers and robots work collaboratively in the chemistry lab using modular and reconfigurable lab equipment interfaced via ROS. A recent paper from Fakhruldeen et al.^78^ shows proof-of-concept toward this direction.

##### Computational Experiment Interface

With the rapid development of computational power and simulation methods, computational experiments are playing a more vital role in catalyst design and optimization,^79^ synthesis planning,^80^ and catalyst discovery.^81^ By using theoretical, fully automated screening methods combining ML and optimization to guide density functional theory (DFT) calculations, Tran and Ulissi^82^ screened across intermetallics for the discovery of electrocatalysts for CO_2_ reduction and H_2_.

The main executor for computational experiments is the high-performance computer (HPC). However, the interaction between the HPC and the coordinator on local computers is different from case to case. The scheduler is the interface for the users on the login nodes to submit batch jobs to the compute nodes on the HPC, as the users cannot run their calculations directly and interactively (as they do on their personal workstations or laptops). The scheduler stores the batch jobs, evaluates their resource requirements and priorities, and distributes the jobs to suitable compute nodes.

There are quite a few open-source scheduling software depending on the setup of HPC, among which SLURM is widely used in research computing services.^83^ Rosen et al.^84^ developed the PyMOFScreen Python package to manage automated DFT calculations, leading to new electronic structure database constructions and accelerating new materials discovery.^85^ Multiple software packages were developed to enable high-throughput screening on the HPC, such as Python Materials Genomics (pymatgen),^86^ FireWorks,^87^ custodian,^86^ Atomate,^88^ GASpy,^81,82^ and ChemEco.^89,90^ Depending on the user’s need as well as the DFT calculation software, the structure and the output file of those Python packages are different and nontransferable. A notable effort in addressing this issue is MolSSI QCArchive,^91^ which offers open access to millions of quantum chemistry calculations done with different software, as well as on-demand computation.

### Current Limitations

Despite the huge improvements made in the literature, a few limitations remain to be overcome before it is possible to achieve a global collaborative network.^11^ The platform-based approach presented heavily relies on the coordinator. This increases the possibility of data loss during transmission, and it will become unsustainable soon with further expansion of the ecosystem. Direct communication between functional components is one potential approach to mitigate this issue, as demonstrated by Fitzpatrick et al.^21^ in letting the planner directly communicate with lab equipment via TCP/IP.

Another limitation is the *ad hoc* data representation and storage. This is particularly important as there is no standard method of representing results or recipes for chemical experiments, despite several competing standards of representing molecules coexisting. The heterogeneous data format lacks interoperability that precludes the full utilization of the embedded information. This problem is further exacerbated when the collaboration between different groups is considered; potentially data generated from one group will be shared and tested on the platform of another group for reproducibility and further experimentations. Moreover, the consequent various data transfer and communication protocols result in low extensibility issues as a considerable amount of time is often required when new hardware or software is integrated, also noted by Breen et al.^92^

Unbalanced chemical data is another limitation to be addressed.^8^ In ML applications, historical data from reaction databases are normally applied as the training set to guide the learning of the planner models. However, only “good” experiment results are published and stored in these databases, limiting the opportunity of learning from “bad” examples,^93^ not to mention those platforms generating experimental data from scratch, without utilizing the prior chemical knowledge at all. A further issue lies in several examples where users are required to manually input chemical data.^42,94^ This is error-prone and limits the potential of full automation.

In brief, improving the interoperability within one platform and between different platforms is a key step in lowering the entry barrier of digitalizing chemistry and promoting a fully automated laboratory. It is thus important for us, as a community, to know how far we are from meeting the prerequisite condition – a fully interconnected data representation capturing the data generated within the experimentation.

## Data Representation and Exchange Protocols

**Figure 2:**
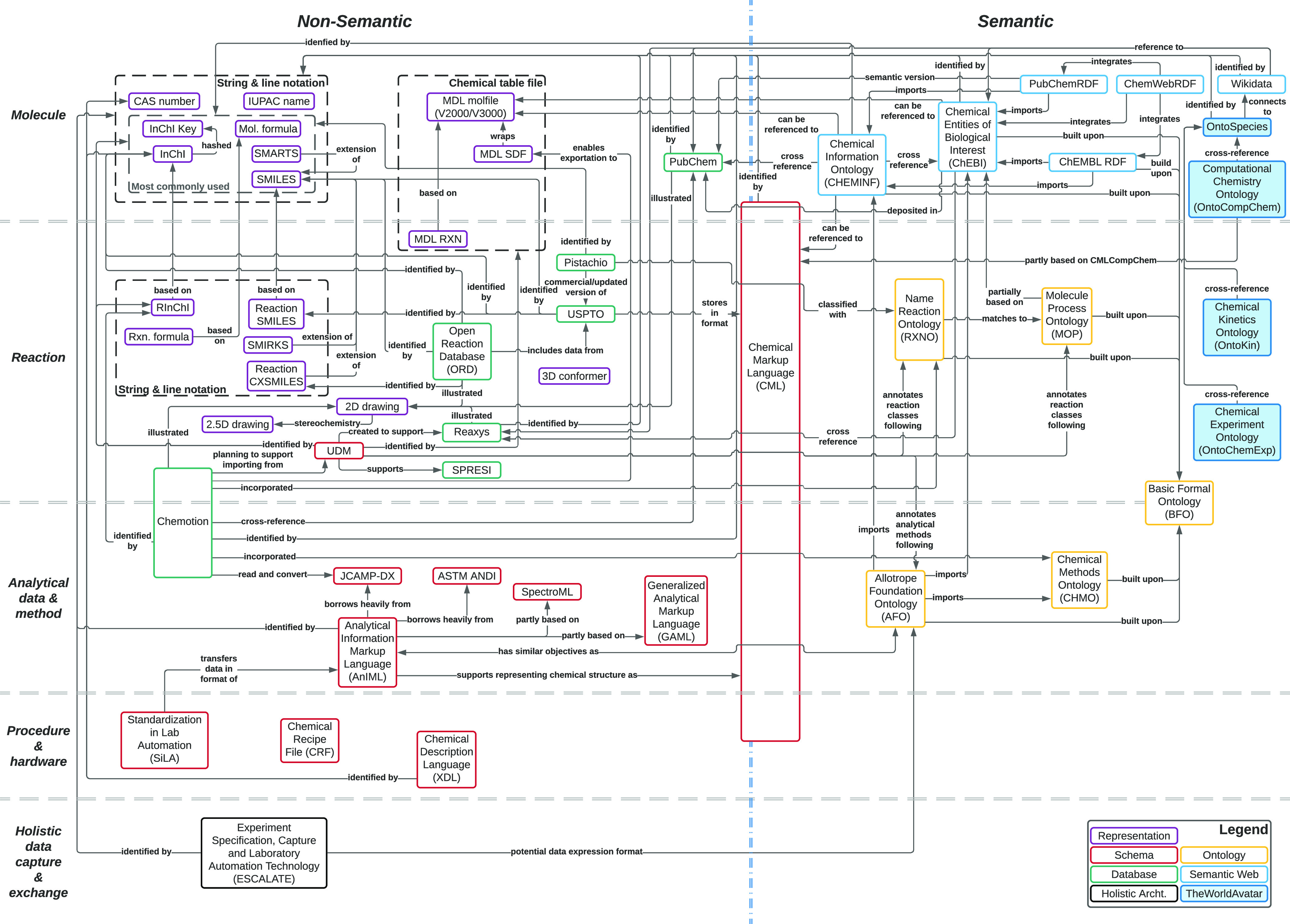
Community landscape toward better data representation and exchange in chemical digitalization. The focus of each category: (a) Molecule: chemical structure, physicochemical properties, and spectral information on a given species; (b) Reaction: chemical reaction scheme, conditions, description of procedures, and statistic summary of the reaction outcome; (c) Analytical data and method: analytical data collected and the methods applied within the experimentation (this is distinct from the spectral information on a given species as this focuses on the data collection process); (d) Procedure and hardware: the operational procedure in an experiment in the format that can be directly executed by hardware; (e) Holistic data capture and exchange: the initiatives to capture all the experimental information generated within the experiment and the exchange of data between different hardware/software. For those on the fence between two categories, we meant they cover both areas. Chemical Markup Language (CML) was labeled as both semantic and non-semantic since it preserves hard-coded and rule-based semantics but not ontologies following semantic web standards.^25^ Basic Formal Ontology (BFO) is an upper-level ontology as the basis of other ontologies, and it does not capture any domain-specific information.

As promoted by various researchers,^1,8,36,95^ the digitalization of chemistry facilitates the collaboration between research groups. Figure 2 reviews data representation and exchange from the different perspectives of a chemical experiment, namely, molecule, reaction, analytical data and method, procedure and hardware, and finally holistic data capture and exchange. Importantly, we distinguish the community efforts into non-semantic and semantic paradigms depending on whether chemical ontologies are involved, and we lay out the connection between them. The agent-based approaches toward standardized and effective communication between each of the components involved are discussed.

### Non-Semantic Representation

In this review, we broadly distinguish non-semantic efforts into four parts: a representation of cheminformatics formats, a schema for constrained encoding of data, a collection of data stored in a database, and finally a holistic architecture that aims to capture all data generated within an experiment.

Since the discovery of the periodic table of the elements, chemical knowledge is built on structures with competing representations.^96^ The most commonly used representation is string and line notation, including SMILES,^97^ InChI,^98^ SMARTS,^99^ SELFIES,^100^ etc. for molecules, and RInChI,^101^ SMIRKS,^102^ etc. for reactions. Chemical table files express molecules and reactions in terms of *x*-*y*-*z* coordinates of atoms and bonds. For a more visual representation, molecules and reactions can be illustrated with 2D line drawings (or 2.5D including stereochemistry), and 3D conformers. These formats are interchangeable with the help of cheminformatics tools, e.g., Open Babel^103^ and RDKit.^104^ An ML application normally starts with encoding structural representations in the form of high-dimensional vectors to map the implicit chemistry to either physicochemical properties of one molecule or reactivity between different molecules.

Popular chemical databases and registry systems normally store various representations of the above with registry numbers, for example, IUPAC name, CAS number, and PubChem CID, for unique and unambiguous identification within themselves and cross-reference between repositories. PubChem^105^ is the largest open-source structural chemical information repository. For reaction informatics,^106^ the scale of open-source databases is much smaller. The USPTO database^107^ is one of the seminal databases in the community and contains 3.7 million reactions extracted from US patents. It was commercialized as Pistachio^108^ containing more than 13 million reactions with annotated reaction classifications using named reaction ontology (RXNO^109^) and expanded coverage to other patent offices, that is, World Intellectual Property Organization (WIPO) and European Patent Office (EPO). Despite the public availability of the USPTO database, its representation schema, Chemical Markup Language (CML) in eXtensible Markup Language (XML), requires extra efforts of format transferring for ML applications. This results in different versions of the USPTO subset that were derived and adapted by various researchers for their applications.^68,110−112^ As the tailored database can be kept private to the research group, it could be difficult for bench-marking new algorithms.

To facilitate the development of ML in chemistry, Open Reaction Database (ORD)^113,114^ was formed to encourage precompetitive data sharing in a standardized format. It records how the reaction was performed, including reaction inputs, conditions, outcome, etc. Notably, ORD uses a protocol buffer as its data structure, instead of the commonly used XML schema. It deliberately avoids the use of ontologies due to insufficient ML applications with ontologies seen in the community.^115^ Despite ORD storing the operation sequence in a machine-readable format, the authors declared it a nongoal at present to make it compatible with programmatic execution on automated synthesis hardware. For more complex operations, ORD only supports a free-text description of the procedure. In terms of the reaction outcome, it focuses more on the statistical summary of the reaction, for example, conversion and yield, and unprocessed analytical data if available. At present, ORD contains 2 million reactions,^115^ including part of the USPTO data set that was converted from CML.

Unified Data Model (UDM)^116^ is another initiative aiming at capturing and integrating the experimental information generated during chemical synthesis. UDM was originally developed by Roche as a transfer model of MDL RD file format for integrating data from various sources into Reaxys database.^117^ It has since evolved to an XML schema with three main elements, namely, *citations*, *molecules*, and *reactions*. In addition to recording the molecule and reaction identifiers, UDM annotates its data with semantic vocabularies. The reaction classification is based on the molecular processes (MOP^118^) and RXNO ontologies, demonstrated by its sample data taken from Reaxys. The analytical method and results type are based on a working draft version of Allotrope Foundation Ontology (AFO^119^) where duplicate entries exist. However, it should be noted that the way UDM integrates the ontologies is by enumerating the ontological classes as a sub-schema of UDM and tagging them to the XML elements as attributes. One general issue with this type of enumeration and attribution is that the relationships declared in the ontologies are not retained in the XML schema, for example, class and subclass relationship between concepts in MOP and RXNO, and the corresponding relationship between result types and analytical methods in the AFO. Looking at the publicly available resources, there are no programmatic constraints over how ontological axioms are enforced in a UDM file. Moreover, UDM allows any type of format for analytical data recording, at least by XML schema itself; tailored tools would be necessary for better utilization of the data. In its latest release, UDM extends its support to the SPRESI database.^120^ Moving forward, UDM aims to provide fully captured representations of reaction predictions and optimizations for multistep reactions. Additional support for environmental health and safety data is also of interest.^121^

Similar to ORD, Chemotion^122^ aims to build a community-driven repository to better publish reaction data generated across different laboratories. In practice, despite containing less data, a key distinguisher of Chemotion is its level of interoperability in enabling programmatic transfer of raw analytical measurements for integration of electronic lab notebook (ELN) from individual laboratories. It does so by supporting reading and converting analytical data in the widely used JCAMP-DX format.^123^ Each published reaction in Chemotion has a semi-machine-readable format with a digital object identifier (DOI). It cross-references compound entries in PubChem. Like UDM, Chemotion incorporates ontologies (RXNO and chemical methods (CHMO^124^)) for semantic annotations at a vocabulary level. On the data validation front, Chemotion automates curation of some types of analytical data, for example, plausibility checks of nuclear magnetic resonance (NMR) data. Human inputs are still required to ensure data quality for publication. To enable more data resources, Chemotion is planning to support reactions stored in a UDM format. Chemotion is also planning to connect ELN to robotics to establish an automated platform for chemical synthesis.^125^

As mentioned, JCAMP-DX is a data standard widely used for recording and sharing analytical data. However, one drawback to its utilization is the lack of validation tools making it difficult for data generated from different software to adhere to the standard terms.^126^ One approach to alleviate this problem is modernizing the standard terms with an XML schema, such as Analytical Information Markup Language (AnIML).^127^ AnIML is partly based on SpectroML^128^ and Generalized Analytical Markup Language (GAML)^126^ and also draws from JCAMP-DX and ASTM ANDI. On the chemical structure side, AnIML supports the CML format together with other commonly used line notations. AnIML aims to provide vendor-neutral analytical and biological data representations that are designed for manufacturers to install and maintain. For the same reason, AnIML provides audit trials and other metadata for reporting information in regulatory processes. At present, AnIML supports most common analytical equipment with detailed documentation for ultraviolet–visible spectrophotometry (UV/vis), chromatography, and indexing.

Up to this point, reviewed efforts are standardizing the data generated during the experiment. Initiatives exist to standardize the instrumentation interface, for example, Standardization in Lab Automation (SiLA).^129^ SiLA is a micro-service architecture using gRPC and HTTP/2 protocols with a protocol buffer as its payload. It adopts a client/server view to describe the devices in the lab environment, where entities expose (multiple) services as SiLA Features accessible to others. SiLA Features are expressed in a predefined XML-based schema and stored in an online repository for service discovery. Each feature is assigned a unique identifier to enable peer-to-peer interactive communication, status queries, and reactions to events. As SiLA is a communication protocol for equipment control, it utilizes AnIML as the medium for the bidirectional transfer of analytical data between laboratory information management systems (LIMS) and chromatography data systems (CDS) in a file-less fashion.^130^ The combination of SiLA and AnIML represents a promising direction: standardized interfaces for instrumentation and unified machine-readable data representations. This results in a complete data package after completion of the analytical experiment, including all the process steps and the generated data.

While SiLA standardizes the equipment interface, chemical recipe file (CRF)^20^ and chemical description language (XDL)^131^ are initiatives to automate experiment execution. They both focus on translating the operational procedures from unstructured descriptions to robot execution commands.

CRF^20^ is a CSV-based schema developed for flow synthesis. Since the instructions are generated based on batch reaction data, human modification is required to enable continuous processes. One notable aspect of their setup is their modularized reaction hardware, making it robotically self-reconfigurable, as demonstrated by the back-to-back synthesis of medicinally relevant small molecules.

XDL^131^ is an XML schema focusing on batch synthesis. It contains three main components as the apparatus to be employed and manually configured, chemicals to be used, and robotic steps abstracted from operations used by chemists in the lab. An ontology is proposed to map the command and hardware executions; however, it is not published in semantic web standards.^25^ Before the instructions are sent to execution, researchers can modify the conditions to benefit human intuitions.

Both CRF and XDL focused on providing a flexible framework to conduct synthesis for multiple molecules. However, neither of them included an automated analysis step. The statistical summary of the chemical synthesis is thus not provided in a standardized format as done by other reaction schemas.

ESCALATE is an attempt toward holistic data capture and exchange.^48^ It proposed an ontological framework for experimentation, supporting data collection, reporting, and experiment generation. This framework captures and reports all the reactions conducted, including “bad reactions”, in line with the cultural change promoted by the community.^95^ In its first release,^48^ the claimed ontological framework was realized by implementing template-based files to store the experimental information, for example, CSV and text files in a file-sharing folder infrastructure (Google Drive). The authors additionally acknowledge that the Allotrope Foundation Data Standard could be incorporated into this data lake. Despite uniform resource locators (URLs) being employed as pointers to some data, the data representation remains heterogeneous and only semi-structured, without the semantic features required by semantic web standards.^25^ In a more recent development,^132^ an ESCALATE REST API^133^ was made available to showcase the possibility of retrieving chemical informatics data from PubChem API, interacting with a Postgres database for submitting experiment jobs to a laboratory, and querying the hosted results.

In general, the non-semantic efforts are closely connected to each other. Multiple representations are normally used within schemas or databases to meet the needs of different applications. Databases cross-reference to each other using registry numbers.

Another notable trend is the adoption of XML schema as data structures. XML is a machine-readable format for algorithmic operations. It relies on string parsing when automating some of the processing steps, for example, the automated unit conversion provided by XDL, where the case-insensitive conversion to a standard unit was performed. However, XML is not designed to host large sets of data as querying between different files can be challenging. The linkage between entries in XML is implicit and requires tailored codes to handle. A solution to this problem could be hosting data in a database and exposing that as the query interface. Yet as demonstrated in the platform-based approach, the same scalability issue would emerge.

It is worth noting the efforts to improve interoperability. Most of the schemas classify items using annotations based on ontological taxonomies. There are also works that claim to have developed ontologies, but that are not however represented in a formal ontology language, such as Web Ontology Language (OWL); their data is still file-based. In the context of this perspective, we consider these outputs to be taxonomies that formalize the hierarchical relationships, distinguishing them from the chemical ontologies that are introduced in the next section. The difficulty of achieving general interoperability remains an issue to be addressed.

### Semantic Representation

Since the landmark publication by Berners-Lee et al.,^24^ the semantic web field has envisioned the next generation of the web in both a human- and machine-readable format for better data sharing among mankind and faster data processing using computers. Through ups and downs, the semantic web community has pivoted from ontologies to linked data, and further to knowledge graphs, which are gaining attention again in recent years. For a comprehensive review of developments in the semantic web field, interested readers are referred to Hitzler.^134^ The focus herein is the uptake of such technologies in the chemistry domain, as illustrated in the right half of Figure 2. For initiatives where only TBox are available, we labeled them as “Ontology”, whereas ABox that are published are labeled “Semantic Web”. Those under “TheWorldAvatar” will be introduced in the next section.

Chemical informatics has a long history of utilizing semantic web technologies. The chemical semantic web^135−137^ is one of such early attempts by Murray-Rust and co-workers, contemporaneously to Berners-Lee’s proposal of the semantic web.^24^ In their work, CML was employed to host the data, prior to OWL becoming the semantic web standard. CML schema covers concepts related to atoms, molecules, computational chemistry, crystallography, spectra, chemical reactions, and polymers. It greatly influenced the development of reaction informatics; especially, it is the molecule representation implicitly used by various cheminformatics software.^138^

Since OWL became more and more popular in modeling ontologies, more activities of ontology development have been demonstrated in the scientific domain. Despite the authors of CML holding the view that ontologies following the semantic web standards^25^ are “too complex for the chemical community to take on board, and provides little effective added value”^139^ compared to their approach, the benefit of semantics motivated the development of chemical ontologies to a great extent, especially work at Royal Society of Chemistry (RSC),^140^ that is, CHMO,^124^ RXNO,^141^ and MOP.^118^ These ontologies are sophisticated and carefully curated. As demonstrated in the non-semantic efforts, they are widely used for annotating reaction classes and analytical methods.

Another driving force of ontology development in the chemistry and biology domain is the European Molecular Biology Laboratory’s European Bioinformatics Institute (EMBL-EBI). In contrast to RSC ontologies that only provide concepts, EBI ontologies provide knowledge at both a terminological and an assertional level, covering small molecules (ChEBI^26^) and cheminformatics (CHEMINF^142^) in a cross-referenced fashion. CHEMINF supports molecular structure representations in the CML format; it also partly transformed data from PubChem into a knowledge base together with cross-reference to their PubChem entries. ChEBI deposited its data in PubChem entries and cross-referenced to Reaxys entries. These ontologies complement other ontologies in the field. For example, CHMO intends to describe the physical and practical methods, whereas CHEMINF covers the computational and theoretical ones.

Ontologizing existing databases was demonstrated in the community, including ChEMBL RDF,^143^ and PubChemRDF,^144^ the semantic version of the current largest open-source chemical information repository, PubChem.^105^ However, the Resource Description Framework (RDF) version of these databases did not come with officially supported SPARQL Protocols and RDF Query Language (SPARQL) endpoint. Galgonek and Vondrášek^145^ recently addressed this issue by integrating PubChem, ChEMBL, and ChEBI data sets as a PostgreSQL database and exposing that to support SPARQL queries. This enabled fast access to chemical data from different sources.

Allotrope Foundation is a collaborative effort from the pharmaceutical industry.^119^ Similar to AnIML, it aims to propose a common data exchange format to unify the laboratory information technology (IT) landscape. It started from realizing the vision of Roberts et al.^146,147^ where an XML schema was envisaged to provide a holistic data format. It later decided to store data based on HDF5 and RDF formats that were controlled by ontologies for semantic capabilities. The foundation now contains three ontologies, namely, AFO, Allotrope Data Format (ADF), and Allotrope Data Model (ADM). AFO is the ontology at the TBox level representing the knowledge in the chemistry domain and it borrows heavily from CHMO. ADF refers to the ontology ABox classified by AFO, extended with more features on data structure and provenance for long-term archiving. ADM is the constraint for how data in ADF should be modeled following AFO. However, only AFO is freely accessible to the public, with the remaining resources restricted to community members.

Compared to non-semantic efforts, a key distinguishing factor of the semantic approach is its fully linked concepts and data instances. This is particularly true for the ontologies reviewed above, as their concepts follow the classification of the Basic Formal Ontology (BFO). The instances stored under each ontology are inherently linked and consistent in logic. This enables interoperability between domains and easy access to data from different sources via SPARQL queries. Moreover, the linked nature made it possible to reduce duplication of information by providing unique identification to the entities, whereas in XML it would be more likely that the same information would appear in different files, for example, when the same molecules are involved in different reactions.

The biology community has demonstrated that the population of data is the key to a broader impact with well-defined ontologies.^148^ However, classifying and annotating data into ontologies while maintaining logical consistency is a challenging task, especially with complex ontologies. It is costly to adopt and creates a high entry barrier. This is reflected in reaction informatics, as ontological data is still very much limited to chemical species information, and there is currently no semantic version of reaction data available. This further exacerbated the problem of insufficient adoption of semantic web technologies in ML and other practical engineering applications, as noted by the developers of ORD,^115^ not to mention that to actually control the equipment execution and automate the data exchange framework is even more challenging. A trade-off between engineering practices and comprehensive representation is thus important. A potential solution to this would be to convert existing databases^149^ into RDF.

The same issue was acknowledged by the Allotrope Foundation^119^ that there is a trend of making simpler data models for practical applications. One of their partner companies, TetraScience, developed an Intermediate Data Schema (IDS)—a JSON-based schema of analytical data as the precursor of the AFO format. Using an agent, data generated from the analytical equipment was collected and converted to ADF for further analytics. Despite being proprietary, it enlightens the way forward to standardize data conversion and integration while it is generated. A perspective from Godfrey et al.^150^ backed this idea, that is, data stored in an ontological framework would very much facilitate the proliferation of interoperable standards and also keep the flexibility of introducing new methodologies.

### Agent-Based Approaches

With the ontological data representation, the way of data generation and consumption is another issue needing to be addressed. By definition, an agent is a piece of “automated” software capable of acting toward achieving its objectives.^151^ In such a process, agents can communicate and coordinate, that is, exchange information with each other, in a standardized format. As aforementioned, TetraScience utilizes agents to standardize data generation; this section focuses on agent applications in standardizing data utilization.

In the context of chemical automation, agent-based approaches can be adapted to replace the functional components within a platform-based approach. Montoya et al.^29^ wrapped different algorithms as agents to suggest the next experiments for DFT calculations on stable materials discovery. Gomes et al.^28^ standardized various tasks as agents (bots) in a platform for crystal-structure phase mapping. Caramelli et al.^30^ applied agent-based model simulations to showcase the effectiveness of multi-threaded networking principles in searching for the optimal solution in the chemical space.

In the above studies, a step was made to turn functional components into modularized agents and standardize the data exchange between them. However, the communication was done by passing in-memory programming variables^28,29^ or posting plain-text on a human messaging platform (Twitter).^30^ As discussed in earlier sections, the same drawbacks such as lack of scalability and interoperability will emerge when scaling up the framework and integrating computational and physical experimentation. A relevant first step toward addressing this issue is demonstrated by DLHub,^152^ which allows users to publish, share, and cite ML models for applications in science.

Following the introduction of ontological data representations, a natural question is to ask whether the use of agents and ontologies can be combined to harness the strengths of both approaches. The challenge of how best to do this has been an open research question since the 2000s.^31^ In theory,^24^ the ontology can help agents with more flexible operations, whereas agents can help the ontology for better data utilization. The Foundation for Intelligent Physical Agents^153^ (FIPA) proposed a set of specifications focusing on communication and interoperability between agents. Specifically, FIPA Ontology Service Specification elaborated the idea of having an ontology agent to support the message interpretation between agents in detail. However, it never made it to the standard stage. In the following years, JADE,^154^ a Java-based software platform that simplifies the implementation of FIPA-compatible multi-agent systems, attempted to provide an ontology in its realization of FIPA standards, but they only provided the ontology as part of the Java code, without connecting to a knowledge base. Attempts to merge the two technologies have been seen in other domains, but not much in chemistry until very recently. An attempt to do this is described in the next section.

## Dynamic Knowledge-Graph-Based Approach

In this section, we explore how a combination of semantic web technologies and multi-agent systems, a dynamic knowledge-graph-based approach, might be applied to realize a complete digital and self-driving laboratory, that is, a chemical digital twin. We review an attempt to develop such an approach in the “World Avatar” project. We subsequently outline a conceptual example of automated closed-loop optimization powered by a dynamic knowledge graph and assess its potential in achieving full automation.

Before diving into further details, we also provide a glossary of terms that are heavily used in this section. We acknowledge that the terms may have different meanings in other contexts; we make no attempt at general definitions here.

**Knowledge graph:** a collection of data and software agents expressed as a directed graph controlled by ontologies, where the nodes and edges refer to concepts and relationships correspondingly. This has broader coverage than the knowledge graph as commonly used in semantic web studies,^134^ where only data are modeled as a directed graph. This is also different from the knowledge graph built based on Reaxys by Segler and Waller^155^ for reaction discovery problems, which expressed molecules as nodes and binary reactions as edges.

**Digital twin:** a virtual replica of real-world entities in the form of a knowledge graph. It is usually created for the real-time monitoring and controlling of real entities and thus should be synchronous with its physical counterpart.

**Autonomous agent:** a semantic web service that acts upon the knowledge graph to achieve predefined goals. Importantly, agents themselves are part of the knowledge graph and represented using the ontology for the agent. While active, agents communicate with each other and interact with the knowledge graph for data retrieval and operation. In the sense of a multi-agent system, the knowledge graph is the “environment” of the agents. The communication between the active agents is conducted via an HTTP request/response. They use ontologies to establish a common understanding of the topic of interest.

**Dynamic knowledge graph:** a knowledge graph that is constantly modified by agents with the latest status of the real world. It controls and influences the real world by updating the specifications of the digital twin and actuating that with agents.

### Current State

The World Avatar (http://theworldavatar.com/) project aims to develop an all-encompassing framework^156^ that is capable of describing any aspect of the world. The World Avatar uses a dynamic knowledge graph, based on an ontological representation of physical entities and interoperable agents. The agents are able to update the knowledge graph with new data, analyze data, make decisions and control entities in the real world. This approach has been suggested to offer a suitable design for a universal digital twin.^157^

Starting from an industrial perspective, the J-Park Simulator, a precursor of the World Avatar, developed a framework that was applied to describe waste energy^158^ and optimize the operation^159^ of an eco-industrial park on Jurong Island, Singapore.^160^

The World Avatar has also been applied to describe a number of different types of chemical data and provides ontologies for quantum chemistry (OntoCompChem^161^), chemical reaction kinetics (OntoKin^162^), chemical species (OntoSpecies^163^), and combustion experiments (OntoChemExp^164^). OntoSpecies links other ontologies to provide unambiguous identification of the chemicals, enabling translation of chemical names when integrating chemical data gathered from different sources.^164^ The ontologies are connected to many of those described in previous sections. For instance, the development of OntoCompChem is partly based on the CompChem terms as described in the CML and the Gainesville Core (GNVC) ontology.^165^ The relationship between these ontologies and other data representations used by the community is shown in Figure 2.

To facilitate the automated data utilization within the knowledge graph, an agent ontology (OntoAgent^166^) was developed as the design pattern of interoperable agents. Each atomic agent is capable of predefined simple tasks with its input/output (I/O) signature linked to the concepts in the domain ontologies. This enabled I/O-based service discoveries to form the agent composition for complex tasks.^166^ Notably, by using OntoAgent to express the agents as part of the knowledge graph, the activities of agents are easily trackable so that provenance can be recorded to document the changes of the knowledge graph over time.

#### Tools and Resources

All outputs from the World Avatar project are available in the public domain. Various agents were developed and released on Github to provide service in the chemistry domain, for example, automated DFT calculations to address inconsistent thermodynamic data,^167^ automated mechanism calibration to improve the alignment between kinetic models and experimental data,^164^ and a question answering system enabling intuitive human data interaction–natural language queries of chemical data covering data from different sources.^168^ Work is in progress to integrate services provided by agents into the natural language processing system so that on-demand computations can be invoked when a question could not be answered with the current knowledge. Users are welcome to check for more functionalities over time: https://kg.cmclinnovations.com/explore/marie.

#### Knowledge Graph Value Proposition

A core strength of the knowledge graph approach is interoperability. The knowledge graph provides a mechanism to combine data, descriptions of software, and hardware interfaces in a standardized way, facilitating automation and allowing communication between agents acting on data from different domains.^164,167^

Another key feature is the open-world assumption, enabling the scalability of a knowledge graph system. Once the skeleton ontology is set, extending knowledge coverage and tailoring against specific applications is easy to manage. It should work just like adding new features to a computational library.

Moreover, once the code of conduct is defined for each of the agents, they can act autonomously and modify the knowledge graph as time elapses. By doing so, the dynamic knowledge-graph reflects and influences the ever-evolving status of the real world.

### Automated Closed-Loop Optimization

The characteristics of dynamic knowledge graphs open up the possibility of a new and powerful approach to closed-loop optimization. In this section, we explore how to apply a dynamic knowledge graph to do this in the context of a case study that was previously automated using a platform-based approach.^42^ The case study considers flow chemistry. However, given suitable ontologies and agents, the underlying principles are expected to generalize to any practices in chemistry where a “design–make–test–analyze” loop is involved.

**Figure 3:**
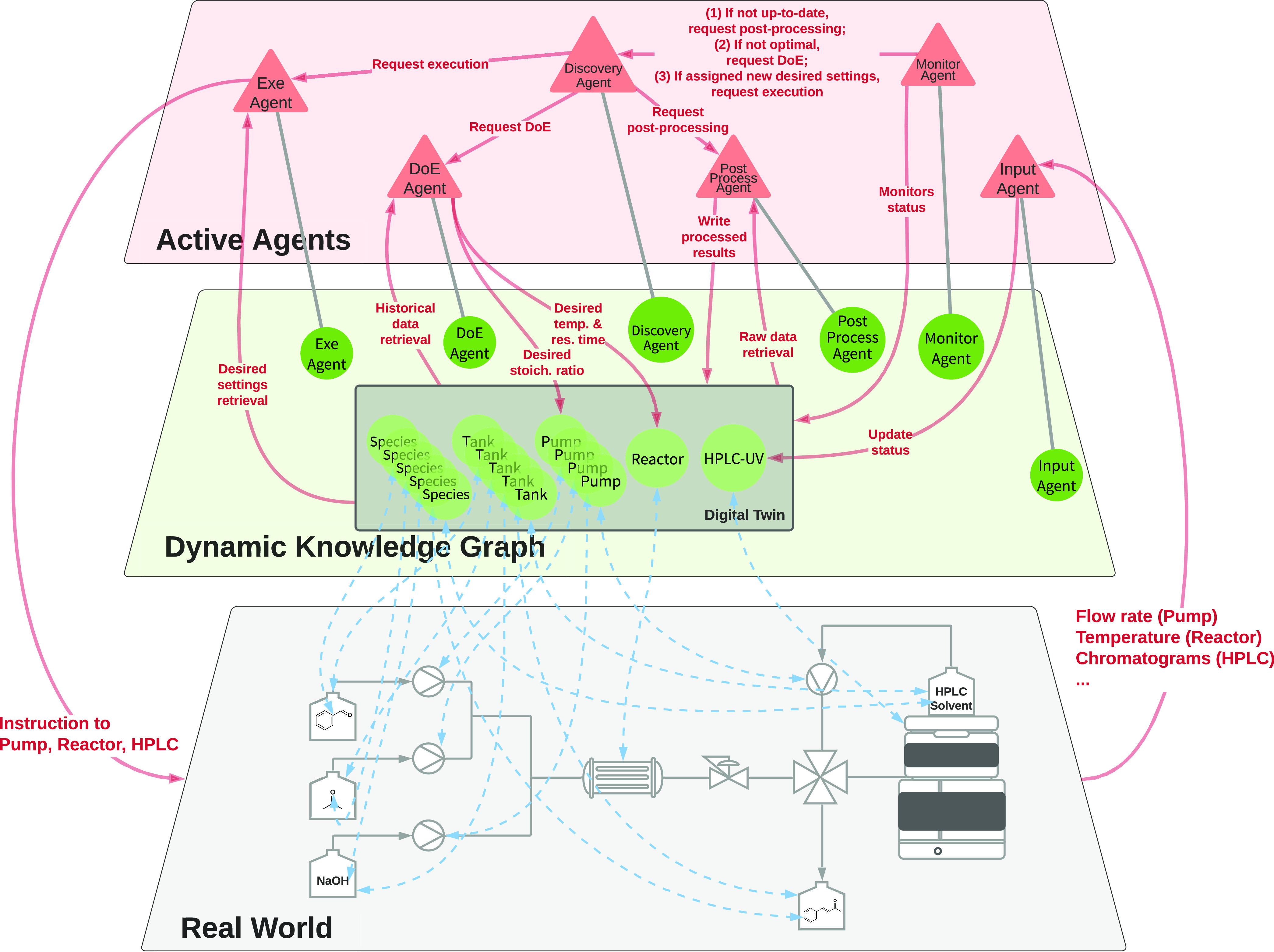
Dynamic knowledge-graph-based approach toward automated closed-loop optimization. The real world layer demonstrates the existing physical entities, adapting from the experimentation setup of Jeraal et al.^42^ The dynamic knowledge graph layer hosts all the data generated during the experimentation and a digital twin of the experimentation apparatus. This layer is dynamic as it reflects and influences the status of the real world in real time. This synchronization is enforced by the agents in the active agents layer, which are instantiated from their ontological representation in the knowledge graph.

Figure 3 illustrates the whole framework consisting of three layers, namely, the real world, the dynamic knowledge graph, and active agents. Reaction data are expressed in ontologies and hosted in the knowledge graph, together with the digital twin of the lab equipment and interoperable agents. Once activated, these agents act autonomously over the knowledge graph and keep the cyber and real worlds synchronized. The update of the digital twin is based on the readings from the equipment. This is not limited to the reaction and analytical equipment but includes environmental sensors located in the laboratory. Each device has its corresponding input agent transmitting the data into the knowledge graph. The monitor agent is responsible for monitoring the status of the digital twin and assessing if further optimization is required. If needed, it invokes the design of experiment (DoE) agent to suggest new experiments and update the configurations of the digital twin. The actuation of such settings is the responsibility of the execution agent to reflect the changes made in the knowledge graph. This loop of self-optimization continues until the monitor agent decides the optimal condition is reached. Importantly, with agents expressed in the OntoAgent format, this framework supports agent discovery service to enable agent-agnostic execution requests.

Compared to the platform-based approach, one distinguishing feature of the dynamic knowledge-graph-based approach is that everything is connected, scalable, unambiguous, distributed, multi-domain, interoperable, accessible, and most importantly evolving in time. As all the digital replicas of the hardware are expressed in the same way, new equipment can be immediately accessed by any existing software once it is instantiated in the knowledge graph. The same applies when adding new ML algorithms wrapped following OntoAgent specifications; standardized interactions with data and HPC services can be established in no time.^167^ This enables the rapid integration of the most advanced algorithms and equipment. Due to the modularized nature, in contrast to heavily intertwined coding logic within a monolithic application, the duty of development of each component is separated, improving the maintainability of the entire system.

Another advantage of this approach is its future-proof nature, for example, its interoperability when integrating with other ontological initiatives in the community. At the species level, OntoSpecies acts like a register system that covers most of the chemical identifiers, making it possible to match with PubChemRDF or other molecular databases. In terms of chemical reactions, OntoKin is already able to describe the kinetic mechanisms of gas-phase chemistry, with OntoChemExp covering the statistical summary of combustion reactions. These concepts can be expanded to describe other chemistry domains of interest. A further opportunity lies in linking the reactions with concepts as defined in RXNO and MOP, embracing their full semantic capabilities. Similar expansion can be made with CHMO or AFO to describe the analytical data and method employed in the experimentation.

### Toward a Digital Laboratory and Beyond

Beyond closed-loop optimization, various researchers have pictured the future toward the next-generation of autonomous laboratories and a global collaborative network.^1,8,11,15,36,40,66,92,146,147^ Jointly, we listed below a few key challenges and how we see the knowledge-graph-based approach helping.

#### Data Generation, Integration, and Sharing

This challenge lies in the data management practice in the platform-based approach.^8,36^ Going toward a full digitalization, the ability to capture all generated data within an experiment (even a “bad” reaction), integrate it with literature data, and share with the community is crucial for navigating in the chemical space. As aforementioned, the knowledge-graph-based approach is designed to be a holistic data capture and exchange framework. With a consensual description of the experiment, literature data stored in the open-source databases can be converted into the ontological format, integrated with the newly generated data.

Roberts et al.^147^ envisioned a combination of XML and relational databases to achieve the same goal. However, the authors acknowledged that a database is difficult for a nonspecialist to explore without clear documentation. To enable data-agnostic queries within the knowledge graph, question answering systems can be of help.^168^ Researchers can thus interact with data intuitively from anywhere at any time, aligning with FAIR principles.^169^ The semantic-rich nature incorporates prior knowledge into the data, presenting the potential to explore informed ML applications.^170^

#### Orchestration of Physical and Computational Experiments

This challenge lies in the emerging trend of physically synthesizing the compounds identified by computational high-throughput screening.^8,65,92,171,172^ In a platform-based approach, this requires a heavy workload on the coordinator to manage the information flow and to orchestrate the software and hardware from different vendors. SiLA and AnIML are the initiatives to provide standardized interfaces and data reporting for proprietary hardware, adopting a mindset of peer-to-peer information exchange that is similar to the platform-based approach.

Whereas in the vision by Roberts et al.^146,147^ and a dynamic knowledge-graph, information is promoted to be accessible to all stakeholders within a laboratory environment, flattening the structural design. For instance, active agents in the World Avatar share the same world-view. The communication between them only serves as a pointer to the correct resources (IRIs). This enables asynchronous communication to accommodate time-consuming activities. Moreover, the communication itself is stored in the knowledge graph and accessible to all agents: everything is transparent and FAIR. By further introducing dependency between different concepts, both data and instructions to the instrument will act like a flow of information traveling in the knowledge graph, analogous to an adaptive organism.

#### Democratization of Chemical Automation

As previously discussed, different approaches toward chemical automation coexist. Choices are to be made for groups upgrading from a common lab environment. Ideally, an off-the-shelf solution should be available that is compatible with any platform to lower the entry barrier. Therefore, interoperability is key toward the democratization of chemical automation.

By design, the knowledge graph approach is able to connect to any laboratory. As it is based on ontologies abstracted from the laboratory entities, it is possible to instantiate a new lab into the knowledge graph and utilize the framework. Developing such a usable and reusable ontology is an iterative process and requires the consensus of the domain. It is envisioned to be a community effort in developing and maintaining its life-cycle. As demonstrated by the general semantic web community^134^ and particular application experience in the chemical engineering community (OntoCAPE^173^), trial-and-error will be inevitable in the coming decade. However, it is reasonable to be positive given the successful adoption of these technologies by giant IT companies.^174^ In that regard, the World Avatar is an open project with all resources available on Github and welcomes contributions from the community.

#### Role of Human Researchers

Despite the advantage of chemical automation, there has been scepticism that the automation of chemistry will replace the bench chemist.^175^ In our view, the development of a digitalized and automated laboratory would enhance the capability of human researchers, enabling them to focus on creative activities, without worrying about the exact physical steps required to achieve their goals. This is similar to how the computer changed our way of working and increased productivity. Since the data in the knowledge graph is easy to query, researchers can focus on interpreting the experimental data and finding insights in historical knowledge generated from mankind.^106,176^ There exists an opportunity for researchers to encode their chemistry intuition into the knowledge graph, essentially making a digital twin of themselves. It would be possible for researchers from different laboratories to exchange views and establish collaborations previously unfeasible. It would be interesting to see what human intuition can achieve when empowered by greater computing abilities.

Moreover, the linked nature of semantic web technologies can bring us further to smart factories, smart buildings, and smart grids,^177^ as has already been demonstrated by the application of the World Avatar in smart city planning,^178^ and the UK Digital Twin^157^ (https://kg.cmclinnovations.com/explore/digital-twin). By constructing a digital laboratory and linking it to the wider context, we believe it will facilitate multi-scale and cross-domain interactions between scientists, engineers, and policy makers to investigate how research done in the lab would affect the whole world. Equipped with scenario analysis, this will help to identify the direction science advances.

## Conclusions and Outlook

This contribution was motivated by the absence of standardized data representations and communication protocols, which precludes further development toward the vision of a global collaborative research network.

We performed a thorough review of the data flow between the different functional components within state-of-the-art studies on chemical automation. We found the common platform-based approach employs *ad hoc* data representations and subsequently different data transfer protocols. This results in scalability issues when integrating new hardware and software, and interoperability issues when collaborating among different platforms: better data representation and exchange are desired.

We reviewed both semantic and non-semantic efforts in the community and outlined the connections between initiatives. Besides the existence of a pattern to promote semantic representations of chemical knowledge, studies are emerging to use agent-based approaches for standardized generation and consumption of data.

With our past experience in closed-loop optimization and knowledge-graph development, we conjecture that a dynamic knowledge-graph-based approach would enable rapid integration of data and AI-based agents for chemical discovery and development. By integrating physical entities into the cyber space, it promotes better utilization of the plethora of computational power in our efforts toward a sustainable future.^179^

In light of the Industry 4.0 revolution, as well as the current COVID situation, this perspective combines the review of common practices in data representation/exchange, community landscape in the development of better data for reaction informatics, and also an outlook toward the holistic integration of automation, AI, and chemistry. The topic of this perspective is timely, and we believe it will start thought-provoking conversations over our way toward fully digitalized chemistry as a community.

Following the knowledge graph approach, hopefully in the not too distant future, we will see the realization of a global collaborative research network. We envisage it would allow more interdisciplinary studies to be conducted for a better understanding of the research activities of mankind. With such further advancements to knowledge graph technology, we are looking forward to a sustainable future in the commencing decade.

## Supporting Information Available

Detailed findings from the selected state-of-the-art studies in chemical automation: To the best of our knowledge, we identified the functional component realization in a platform-based approach in Table S1 and in Table S2, we categorized the data flow and communication protocols between the functional components following the method described in the main text (PDF)
