# Supplementary material for: From Platform to Knowledge Graph: Evolution of Laboratory Automation
Source: JACS Au. 2022 Jan 10;2(2):292–309. doi: 10.1021/jacsau.1c00438 (PMC8889618; doi:10.1021/jacsau.1c00438)
Supplement: Supplementary file 1 — au1c00438_si_001.pdf [file au1c00438_si_001.pdf]

From Platform to Knowledge Graph: Evolution of Laboratory Automation

Jiaru Bai,[†,‖] Liwei Cao,[†,‖] Sebastian Mosbach,[†,‡] Jethro Akroyd,[†,‡]

Alexei A. Lapkin,[*,†,‡] and Markus Kraft[*,†,‡,¶,§]

†*Department of Chemical Engineering and Biotechnology, University of Cambridge, Philippa Fawcett Drive, Cambridge CB3 0AS, United Kingdom*

‡*Cambridge Centre for Advanced Research and Education in Singapore (CARES), CREATE Tower #05-05, 1 Create Way, Singapore 138602*

¶*School of Chemical and Biomedical Engineering, Nanyang Technological University, 62 Nanyang Drive, Singapore 637459*

§*The Alan Turing Institute, London NW1 2DB, United Kingdom*

‖*J.B. and L.C. contributed equally to this work.*

E-mail: aal35@cam.ac.uk; mk306@cam.ac.uk

Phone: +44 (0)1223 762784

Supporting Information Available

This file lists the detailed findings from the selected state-of-the-art studies in chemical automation. To the best of our knowledge, we identified the functional component realisation in a platform-based approach in Table S1. Besides, in Table S2, we categorised the data flow and communication protocols between the functional components following the method described in the main text.

S1 Supporting Information

Table S1: Functional component realisation of selected state-of-the-art studies in chemical automation. For computational model development applications, the model generated as planner was trained on executor with user-defined optimisers. The executors are physical hardware unless otherwise stated. HPC: high-performance computing. MS: mass spectrometer. IR: infrared spectroscopy. BPR: back pressure regulator. HPLC: high-performance liquid chromatography. NMD-M3: Nanotechnology Materials Data Mining, Modeling & Management. VASP: Vienna *ab initio* Simulation Package. ASE: Atomic Simulation Environment. ICSD: Inorganic Crystal Structure Database.

**Reference**	**Application**	**System**	**Receptionist**	**Coordinator**	**Librarian**	**Planner**	**Executor**
Fitzpatrick et al. [1]	Reaction condition optimisation of a three-dimensional heterogeneous catalytic reaction and a five-dimensional Appel reaction	Flow reactor	Web browser with credentials	LeyLab: PHP-based	Database	Complex Method [2]	Vapourtec R2/R4, online MS, webcam, and inline IR
Ingham et al. [3]	Multi-step synthesis of 2-aminoadamantane-2-carboxylic acid	Flow reactor	Web browser	Octopus: Python-based	CSV file	N/A	Uniqsis, Vapourtec R2+, Knauer machine (HPLC pump), and webcams
Fitzpatrick et al. [4]	Reaction condition optimisation for three active pharmaceutical ingredients (APIs)	Flow reactor	Web browser	LeyLab: PHP-based	Database: MySQL	Complex Method [2]	Flow reactor, HPLC, inline IR, BPR, computer vision module, *etc.*
Nikolaev et al. [5]	Automated designing, executing, and evaluating carbon nanotube growth experiments	Batch reactor	NMD-M3 software	NMD-M3 software	Database within NMD-M3 software	Random forest and genetic algorithm	In-house built ARES platform: inverted Raman microscope, laser, pressure gauge, vaccum pump and gas mass glow controller

Table S1: (Continued)

Reference	Application	System	Receptionist	Coordinator	Librarian	Planner	Executor
Wigley et al.[6]	Process condition optimisation of the production of Bose-Einstein condensates (BEC)	Exponential evaporation ramp	N/A	Python code	MAT file	MLOO:[6] Gaussian process-based	Cooling ramp, evaporation process
Greenaway et al.[7]	Automated porous organic cages discovery by high-throughput screening	Batch reactor	Chemspeed software	Python code	CSV from Chemspeed	Predefined algorithmic workflow in Fig. 6 in Greenaway et al.[7]	**Computational:** HPC; **Physical:** Chemspeed Accelerator SLT-100 automated synthesis platform, NMR, HRMS, HPLC, *etc.*
Bédard et al.[8]	High-yielding implementations of C-C and C-N cross-coupling, olefination, reductive amination, nucleophilic aromatic substitution (S_NAr), photoredox catalysis, and a multistep sequence	Flow reactor	LabVIEW-based GUI	Matlab code	MAT file	SNOBFIT algorithm	Reactor, pump, pressure sensor, flow meter, phase sensor, IR based temperature sensor, camera, HPLC, IR, raman spectroscopy, MS
Caramelli et al.[9]	Collaborative chemical space exploration by two internet-connected robots on multiple chemical processes	Batch reactor	N/A	Python code	Twitter feed	Random search, grid search, and Monte-Carlo	Peristaltic pumps, webcam, and reaction flask
Skilton et al.[10]	Etherification of n-propanol in supercritical CO_2 over a γ-Al_2O_3 catalyst, optimized for the formation of di-n-propyl ether	Flow reactor	GLC Solutions	GLC Solutions	Stored within GLC Solutions	GLC Solutions software	Require local human operator for filling stock

Table S1: (Continued)

Reference	Application	System	Receptionist	Coordinator	Librarian	Planner	Executor
Coley et al.[11]	Automated synthesis of 15 medicinal relevant small molecules	Flow reactor	Web browser (for ASKCOS) and Python-based GUI (for robotic execution)	Human researcher	Database (USPTO and Reaxys)	ASKCOS package askcos.mit.edu	Modularised flow reactor, six axis UR3 Universal Robot, BPR, MS, HPLC, NMR, *etc.*
Roch et al.[12]	Automated chemical recipe discovery	Batch reactor	NLP-based chatbot (Twitter, Slack, Gmail)	ChemOS: Python-based	SQLite database	Random search, Spearmint, SMAC, and Phoenics[12]	Various lab equipment based on user needs: pumps, HPLC, *etc.*
Montoya et al.[13]	End-to-end computational system for autonomous materials discovery	Binary/ternary inorganic chemicals	N/A	Python code	JSON	Algorithms in Fig. 2 in Montoya et al.[13]	**Computational:** DFT simulation on AWS EC2
Li et al.[14]	Discovery of optically active chiral inorganic perovskite nanocrystals	Flow reactor	Web browser with SSH login and Python-based GUI	MAOSIC: Python-based	DBMS, *e.g.* MySQL	SNOBFIT	Microfludic reactor, collaborative robot, syringe pump, environment sensor, *etc.*

Table S1: (Continued)

Reference	Application	System	Receptionist	Coordinator	Librarian	Planner	Executor
Xue et al.[15]	Materials discovery for very low thermal hysteresis (ΔT) multicomponent NiTi-based shape memory alloys	Batch reactor	N/A	Python code	CSV (based on supplementary information)	Efficient global optimisation and knowledge gradient	**Computational:** QUANTUM ESPRESSO planewave pseudopotential package; **Physical:** synthesis performed manually
Cao et al.[16]	Formulated product recipe optimisation	Batch reactor	N/A	Python code	CSV and textfile	TSEMO[17]	Robotic platform, syringe pumps, pH analyser, turbidity analyser, and viscometer
Jeraal et al.[18]	Multi-objective reaction condition optimisation for aldol condensation reaction	Flow reactor	Matlab-based GUI	Matlab application	CSV file	TSEMO[17]	Vapourtec R2/R4, Agilent 1260 HPLC, and BPR
King et al.[19]	Identification of genes encoding orphan enzymes in yeast *Saccharomyces cerevisiae*	Mobile robotic platform	N/A	Adam: robot scientist	KEGG (prior), MySQL database (new)	Bioinformatic methods (hypotheses generation); two-factor DoE	Freezer, liquid handler, incubators, *etc.*
King et al.[20]	Functional genomics synthesis optimisation for aromatic amino acid synthesis pathway in yeast	Mobile robotic platform	N/A	Laboratory Information Management System	Database within LIMS	Bayesian analysis of decision-tree learning	Liquid handling, pipetting and mixing liquids on microtitre plates
Ingham et al.[21]	Multi-step reaction condition optimisation for pyrazine-2-carboxamide and piperazine-2-carboxamide	Flow reactor	Web browser	Octopus: Python-based	CSV file	Complex Method[2]	Vapourtec R2+/R4, HPLC, computer vision module, *etc.*

Table S1: (Continued)

Reference	Application	System	Receptionist	Coordinator	Librarian	Planner	Executor
Schweidtmann et al.[22]	Multi-objective reaction condition optimisation for S_NAr reaction and N-benzylation	Flow reactor	N/A	Matlab code	CSV file	TSEMO[17]	JASCO PU980 pumps, Vapourtec, Agilent 1100 HPLC, and BPR
HamediRad et al.[23]	Biosynthetic pathway optimisation of lycopene	Continuous workflow	N/A	BioAutomata: Python-based	DAT file	Bayesian optimisation	iBioFAB automated platform[24]
MacLeod et al.[25]	Self-driving laboratory for accelerated discovery of thin-film materials	Mobile robotic platform	Python-based GUI	ChemOS: Python-based	Database	Phoenics within ChemOS	North Robotics N9 robots and liquid handler
Segler et al.[26]	Computational model development for synthesis planning of small molecules	Single-step reactions	N/A	Python code	Extracted from ZINC and Reaxys	Neural network combined with Monte Carlo tree search	**Computational:** single NVIDIA K80 graphics processing unit
Steiner et al.[27]	Automated synthesis of three pharmaceutical compounds: diphenhydramine hydrochloride, rufinamide, and sildenafil	Batch reactor	N/A	Chemputer: Python-based	N/A	Synthesis route set by human	Reactor, pumps, filter, separator, and rotary evaporator
Mehr et al.[28]	Automated syntheses of 12 compounds from the literature, including the analgesic lidocaine, the Dess-Martin periodinane oxidation reagent, and the fluorinating agent AlkylFluor	Batch reactor	Web browser (ChemIDE), allow editing proposed synthesis steps	Chemputer: Python-based	Literature operation procedure in textfile (free-text format)	SynthReader: NLP-based synthesis action sequence planner	Reactor, pumps, filter, separator, rotary evaporator, vacuum, stirrer, conductivity sensor, heater *etc.*

Table S1: (Continued)

Reference	Application	System	Receptionist	Coordinator	Librarian	Planner	Executor
Kusne et al.[29]	Automated phase mapping and property optimisation for accelerating materials discovery with high-throughput X-ray diffraction	Ge–Sb–Te ternary system	GUI (details not provided)	CAMEO: Matlab-based	Preloaded database (ICSD and AFLOW.org)	Bayesian-based active learning method	Not implemented yet
Burger et al.[30]	Photocatalysts material discovery for hydrogen production from water	Mobile robotic chemist	Java-based GUI	Java-based	CSV file	Bayesian optimiser[31]	KUKA mobile robot to conduct experiments workflow in the lab, *e.g.*, gas chromatograph measurements, solid dispensing, *etc.*
Tran and Ulissi[32]	Computational screening for electrocatalysts discovery of CO_2 reduction and H_2 evolution	Electrochemical reduction	N/A	Python code	MongoDB database	ML method in TPOT package[33]	**Computational:** High-throughput DFT by VASP using ASE on HPC
Christensen et al.[34]	Autonomous process optimisation of a palladium-catalysed stereoselective Suzuki-Miyaura coupling	Batch reactor	N/A	ChemOS: Python-based	Database	Phoenics and Gryffin within ChemOS	Chemspeed SWING robotic system, Agilent 1100
Gao et al.[35]	Computational model development for optimal reaction condition recommendation of organic synthesis reactions	Single-product and single-step reactions	N/A	Python code	Reaxys database	Nerual Network based condiction prediction tool	**Computational:** single NVIDIA GeForce GTX 1080 GPU

Table S1: (Continued)

Reference	Application	System	Receptionist	Coordinator	Librarian	Planner	Executor
Rosen et al.[36]	Accelerating chemical space exploration of metal–organic frameworks with quantum-chemical calculations and machine learning	Crystalline solids	N/A	Python code	Database	Crystal graph convolutional neural network (CGCNN)	**Computational:** high-throughput DFT by VASP using ASE on HPC
Taylor et al.[37]	Automated determination of reaction models and kinetic parameters	Flow reactor	Matlab-based GUI	Matlab code	Reaction model database in Matlab	MILP optimising reaction kinetics	Tubular reaction vessel built in-house, HPLC pumps, auto-sampler, and HPLC
Waldron et al.[38]	Rapid kinetic model identification	Flow reactor	LabVIEW-based GUI	Python code	CSV file	MBDoE algorithm	Flow reactor, pumps, sampler-dilutor, and HPLC

Table S2: Data flow and communication protocols between functional components of the selected state-of-the-art studies in chemical automation. The workflow indicates the data flow exchanged within the platform that managed by the coordinator. EP: executor (physical). EC: executor (computational). MS: mass spectrometer. IR: infrared spectroscopy. NMD-M3: Nanotechnology Materials Data Mining, Modeling & Management. HPC: high-performance computing. CRF: chemical recipe file. XDL: chemical description language. VASP: Vienna *ab initio* Simulation Package. ASE: Atomic Simulation Environment.

**Reference**	**Receptionist - Coordinator**	**Coordinator - Librarian**	**Coordinator - Planner**	**Coordinator - Executor**	**Inner Executor (physical)**	**Inner Executor (computational)**	**Workflow (R C L P EP EC)**
Fitzpatrick et al. [1]	TCP/IP	MySQL database query (TCP/IP)	In-memory cache	TCP-IP: MS readings transmitted by Arduino-based analogue to serial converter (RS232 serial communication to Ethernet); webcam liquid level position computed by Raspberry Pi in JSON format	Arduino and Raspberry Pi worked as interface for controlling the equipment	N/A	C-[R-P-L-EP-P]
Ingham et al. [3]	HTTP	TCP/IP	Python variables	(1) USB root hub: two webcams; (2) TCP/IP: Uniqsis, Knauer machines and Vapourtec were connected via Brainboxes ES-701 and ES-257 ethernet-to-serial adapters	Raspberry Pi worked as interface for controlling the equipment	N/A	C-[R-P-L-EP-P]

Table S2: (Continued)

Reference	Receptionist - Coordinator	Coordinator - Librarian	Coordinator - Planner	Coordinator - Executor	Inner Executor (physical)	Inner Executor (computational)	Workflow (R C L P EP EC)
Fitzpatrick et al.[4]	HTTPS	Database query (TCP/IP)	In-memory cache	TCP/IP (with RS232 serial to Ethernet adaptor): equipment was placed within a VLAN that connected to LeyVM server via SSH tunnel	Raspberry Pi worked as interface for controlling the equipment	N/A	C-[R-P-EP-L-P]
Nikolaev et al.[5]	Handled by NMD-M3 software	Handled by NMD-M3 software	Handled by NMD-M3 software	Handled by NMD-M3 software	Through in house built software in C#/.NET	N/A	C-[R-P-EP-L-P]
Wigley et al.[6]	N/A	File transfer	Python variables	File transfer: MAT and textfile	Not specified	N/A	C-[L-P-EP-L]
Greenaway et al.[7]	Within Chemspeed software	File transfer	SSH to HPC	Set the input variables through Chemspeed software	Controlled through Chemspeed software	N/A	C-[EC-L-R-P-EP]
Bédard et al.[8]	Matlab variables	Matlab varialbes	Matlab variable	LabVIEW: through national instrument, serial modem cable, USB cable	Serial command through LabVIEW	N/A	R-P-EP-L-P
Caramelli et al.[9]	N/A	Plaintext as Python variables	Python variables	Python variables handled by gpio and opencv Python libraries	Pumps and webcam are interfaced via pcDuino3 board	N/A	C-[L-P-EP-L]
Skilton et al.[10]	Passed within GLC Solutions	Passed within GLC Solutions	Passed within GLC Solutions	Remote computer control through GLC Solutions	Handled by GLC Solutions	N/A	C-[L-P-E-L]

Table S2: (Continued)

**Reference**	**Receptionist - Coordinator**	**Coordinator - Librarian**	**Coordinator - Planner**	**Coordinator - Executor**	**Inner Executor (physical)**	**Inner Executor (computational)**	**Workflow (R C L P EP EC)**
Coley et al.[11]	CRF file transfer by human researcher	MongoDB database query	Planner generates CRF file to be modified by human researcher	Human researcher pass the CRF file to robotic execution GUI	Universal process bays provide sealing and alignment mechanisms for the fluidic, electrical, and pneumatic process connections	SLURM scheduling software	C-[R(of EC)-L-P(resulted from EC)-R(of EP)-EP]
Roch et al.[12]	JSON, parsed by Python	Database query	Python array	Python pickle object	Raspberry Pi as controller of pumping system, communicated via SCP with the executor codes; Dropbox for synchronising the characterisation equipment	N/A	C-[R-L-P-EP-L]
Montoya et al.[13]	N/A	Python variables	Python variables	Python variables	N/A	AWS Batch API	C-[L-P-EC-L]
Li et al.[14]	TLS encrypted file transfer	SQLAlchemy database query	Python variables (within MAOSIC)	JSON-RPC	Interfaced via high-level and low-level instructions based on JSON-RPC2.0 protocol	N/A	C-[R-L-P-EP-L]
Xue et al.[15]	N/A	Not specified	Python variables	File transfer for DFT	Synthesis performed manually	Handled by Quantum ESPRESSO package	C-[L-P-EC-L]

Table S2: (Continued)

Reference	Receptionist - Coordinator	Coordinator - Librarian	Coordinator - Planner	Coordinator - Executor	Inner Executor (physical)	Inner Executor (computational)	Workflow (R C L P EP EC)
Cao et al.[16]	N/A	File transfer	File transfer	File transfer	File transfer	N/A	C-[L-P-EP-L]
Jeraal et al.[18]	In-memory cache of Matlab variables	File transfer	Matlab variables	File transfer: CSV file	Interfaced via FlowCommander, a software provided by Vapourtec	N/A	C-[R-L-P-EP-L]
King et al.[19]	N/A	Database query	Not specified	File transfer: LABORS (OWL-DL format)	Closed-source software from Caliper Life Sciences	N/A	C-[L-P-EP-L]
King et al.[20]	N/A	Database query	Not specified	Prolog commands through TCP/IP	Robot operations controlled by tool command language translated from Prolog commands	N/A	C-[L-P-EP-L]
Ingham et al.[21]	HTTP	TCP/IP	Python variables	(1) USB root hub: two webcams; (2) TCP/IP: Uniqsis, Knauer machines and Vapourtec were connected via Brainboxes ES-701 and ES-257 ethernet-to-serial adapters	Raspberry Pi worked as interface for controlling the equipments	N/A	C-[L-P-EP-L]

Table S2: (Continued)

Reference	Receptionist - Coordinator	Coordinator - Librarian	Coordinator - Planner	Coordinator - Executor	Inner Executor (physical)	Inner Executor (computational)	Workflow (R C L P EP EC)
Schweidtmann et al.[22]	N/A	File transfer	File transfer: CSV file	File transfer: CSV file	Interfaced via FlowCommander, a software developed by Vapourtec	N/A	C-[EP-L-P-EP-L]
HamediRad et al.[23]	N/A	File transfer	Python variables	File transfer: CSV file (iScheduler code)	Managed by iScheduler scheduling software on iBioFAB platform	N/A	C-[L-P-EP-L]
MacLeod et al.[25]	Python variables	Database query	Python variables	Python variables	Driven by North Robotics C9 controller, which also provides auxiliary controls for third-party instruments and components used by the robot	N/A	C-[R-L-P-EP-L]
Segler et al.[26]	N/A	Python variables	Python variables	Python variables	N/A	Theano-backend Keras	C-[L-P-EC]
Steiner et al.[27]	N/A	N/A	XDL (XML-based file) for synthesis route	XDL file	Arduino as micro-controller	N/A	C-[P-EP]
Mehr et al.[28]	In-memory cache & XDL	Textfile	XDL file	XDL file	Arduino as micro-controller	N/A	C-[R-L-R-EP]

Table S2: (Continued)

**Reference**	**Receptionist - Coordinator**	**Coordinator - Librarian**	**Coordinator - Planner**	**Coordinator - Executor**	**Inner Executor (physical)**	**Inner Executor (computational)**	**Workflow (R C L P EP EC)**
Kusne et al. [29]	Not specified	File transfer: MAT file	Matlab variables	Programmatically generated script via SPEC for the SLAC high-throughput system or a GADDS script for the Bruker system	Not specified	N/A	C-[L-P-EP-L]
Burger et al. [30]	N/A	CSV read/write	File transfer	Various communication protocols (TCP/IP over WIFI/LAN; RS-232)	Simultaneous localisation and mapping (SLAM) was used for robot allocation; Arduino designed as micro-controller	N/A	C-[L-P-EP-L]
Tran and Ulissi [32]	N/A	Database query	Python variables	Atom object converted from JSON	N/A	Managed by Luigi and Fireworks	C-[L-P-EC-L]
Christensen et al. [34]	N/A	Database query	Python variables	File transfer (done by a Python script): CSV, textfile & Python pickle object	Chemspeed AutoSuite acts as the control interface	N/A	C-[R-L-P-EP-L]
Gao et al. [35]	N/A	Reaxys API	Python variables	Python variables	N/A	Theano-backend Keras	C-[L-P-EC-L]
Rosen et al. [36]	N/A	Python variables	Python variables	File transfer (ASE argument to VASP)	N/A	Managed by PyMOFScreen	C-[L-P-EC-L]

Table S2: (Continued)

Reference	Receptionist - Coordinator	Coordinator - Librarian	Coordinator - Planner	Coordinator - Executor	Inner Executor (physical)	Inner Executor (computational)	Workflow (R C L P EP EC)
Taylor et al.[37]	Matlab variables	Matlab variables	Matlab variables	Matlab variables	Matlab variables	N/A	C-[L-P-EP-L]
Waldron et al.[38]	Python variables	File transfer	Python variables	Python variables	LabVIEW acts as the interface	N/A	C-[R-L-P-EP-L]

References

(1) Fitzpatrick, D. E.; Battilocchio, C.; Ley, S. V. A Novel Internet-Based Reaction Monitoring, Control and Autonomous Self-Optimization Platform for Chemical Synthesis. *Org. Process Res. Dev.* **2016**, *20*, 386–394.

(2) Kazmierczak Jr, R. F. Optimizing Complex Bioeconomic Simulations Using an Efficient Search Heuristic. *DAE Research Report No. 704C61* **1996**, 1–38.

(3) Ingham, R. J.; Battilocchio, C.; Fitzpatrick, D. E.; Sliwinski, E.; Hawkins, J. M.; Ley, S. V. A Systems Approach Towards an Intelligent and Self-Controlling Platform for Integrated Continuous Reaction Sequences. *Angew. Chem., Int. Ed.* **2015**, *127*, 146–150.

(4) Fitzpatrick, D. E.; Maujean, T.; Evans, A. C.; Ley, S. V. Across-the-World Automated Optimization and Continuous-Flow Synthesis of Pharmaceutical Agents Operating through a Cloud-Based Server. *Angew. Chem., Int. Ed.* **2018**, *57*, 15128–15132.

(5) Nikolaev, P.; Hooper, D.; Webber, F.; Rao, R.; Decker, K.; Krein, M.; Poleski, J.; Barto, R.; Maruyama, B. Autonomy in Materials Research: A Case Study in Carbon Nanotube Growth. *npj Comput. Mater.* **2016**, *2*, 1–6.

(6) Wigley, P. B.; Everitt, P. J.; van den Hengel, A.; Bastian, J. W.; Sooriyabandara, M. A.; McDonald, G. D.; Hardman, K. S.; Quinlivan, C. D.; Manju, P.; Kuhn, C. C. N.; Petersen, I. R.; Luiten, A. N.; Hope, J. J.; Robins, N. P.; Hush, M. R. Fast Machine-Learning Online Optimization of Ultra-Cold-Atom Experiments. *Sci. Rep.* **2016**, *6*, 1–6.

(7) Greenaway, R. L.; Santolini, V.; Bennison, M. J.; Alston, B. M.; Pugh, C. J.; Little, M. A.; Miklitz, M.; Eden-Rump, E. G. B.; Clowes, R.; Shakil, A.; Cuthbertson, H. J.; Armstrong, H.; Briggs, M. E.; Jelfs, K. E.; Cooper, A. I. High-Throughput

Discovery of Organic Cages and Catenanes Using Computational Screening Fused with Robotic Synthesis. *Nat. Commun.* **2018**, *9*, 1–11.

(8) Bédard, A.-C.; Adamo, A.; Aroh, K. C.; Russell, M. G.; Bedermann, A. A.; Torosian, J.; Yue, B.; Jensen, K. F.; Jamison, T. F. Reconfigurable System for Automated Optimization of Diverse Chemical Reactions. *Science* **2018**, *361*, 1220–1225.

(9) Caramelli, D.; Salley, D.; Henson, A.; Camarasa, G. A.; Sharabi, S.; Keenan, G.; Cronin, L. Networking Chemical Robots for Reaction Multitasking. *Nat. Commun.* **2018**, *9*, 1–10.

(10) Skilton, R. A. et al. Remote-Controlled Experiments with Cloud Chemistry. *Nat. Chem.* **2015**, *7*, 1–5.

(11) Coley, C. W. et al. A Robotic Platform for Flow Synthesis of Organic Compounds Informed by AI Planning. *Science* **2019**, *365*, eaax1566.

(12) Roch, L. M.; Häse, F.; Kreisbeck, C.; Tamayo-Mendoza, T.; Yunker, L. P. E.; Hein, J. E.; Aspuru-Guzik, A. ChemOS: An Orchestration Software to Democratize Autonomous Discovery. *PLoS One* **2020**, *15*, e0229862.

(13) Montoya, J. H.; Winther, K. T.; Flores, R. A.; Bligaard, T.; Hummelshøj, J. S.; Aykol, M. Autonomous Intelligent Agents for Accelerated Materials Discovery. *Chem. Sci.* **2020**, *11*, 8517–8532.

(14) Li, J.; Li, J.; Liu, R.; Tu, Y.; Li, Y.; Cheng, J.; He, T.; Zhu, X. Autonomous Discovery of Optically Active Chiral Inorganic Perovskite Nanocrystals through an Intelligent Cloud Lab. *Nat. Commun.* **2020**, *11*, 1–10.

(15) Xue, D.; Balachandran, P. V.; Hogden, J.; Theiler, J.; Xue, D.; Lookman, T. Accelerated Search for Materials with Targeted Properties by Adaptive Design. *Nat. Commun.* **2016**, *7*, 1–9.

(16) Cao, L.; Russo, D.; Felton, K.; Salley, D.; Sharma, A.; Keenan, G.; Mauer, W.; Gao, H.; Cronin, L.; Lapkin, A. A. Optimization of Formulations Using Robotic Experiments Driven by Machine Learning DoE. *Cell Rep. Phys. Sci.* **2021**, *2*, 100295.

(17) Bradford, E.; Schweidtmann, A. M.; Lapkin, A. Efficient Multiobjective Optimization Employing Gaussian Processes, Spectral Sampling and A Genetic Algorithm. *J. Glob. Optim.* **2018**, *71*, 407–438.

(18) Jeraal, M. I.; Sung, S.; Lapkin, A. A. A Machine Learning-Enabled Autonomous Flow Chemistry Platform for Process Optimization of Multiple Reaction Metrics. *Chem. Methods* **2021**, *1*, 71–77.

(19) King, R. D.; Rowland, J.; Oliver, S. G.; Young, M.; Aubrey, W.; Byrne, E.; Liakata, M.; Markham, M.; Pir, P.; Soldatova, L. N.; Sparkes, A.; Whelan, K. E.; Clare, A. The Automation of Science. *Science* **2009**, *324*, 85–89.

(20) King, R. D.; Whelan, K. E.; Jones, F. M.; Reiser, P. G. K.; Bryant, C. H.; Muggleton, S. H.; Kell, D. B.; Oliver, S. G. Functional Genomic Hypothesis Generation and Experimentation by a Robot Scientist. *Nature* **2004**, *427*, 247–252.

(21) Ingham, R. J.; Battilocchio, C.; Hawkins, J. M.; Ley, S. V. Integration of Enabling Methods for the Automated Flow Preparation of Piperazine-2-Carboxamide. *Beilstein J. Org. Chem.* **2014**, *10*, 641–652.

(22) Schweidtmann, A. M.; Clayton, A. D.; Holmes, N.; Bradford, E.; Bourne, R. A.; Lapkin, A. A. Machine Learning Meets Continuous Flow Chemistry: Automated Optimization Towards the Pareto Front of Multiple Objectives. *Chem. Eng. J.* **2018**, *352*, 277–282.

(23) HamediRad, M.; Chao, R.; Weisberg, S.; Lian, J.; Sinha, S.; Zhao, H. Towards a Fully Automated Algorithm Driven Platform for Biosystems Design. *Nat. Commun.* **2019**, *10*, 1–10.

(24) Chao, R.; Yuan, Y.; Zhao, H. Building Biological Foundries for Next-Generation Synthetic Biology. *Sci. China: Life Sci.* **2015**, *58*, 658–665.

(25) MacLeod, B. P. et al. Self-Driving Laboratory for Accelerated Discovery of Thin-Film Materials. *Sci. Adv.* **2020**, *6*, eaaz8867.

(26) Segler, M. H. S.; Preuss, M.; Waller, M. P. Planning Chemical Syntheses with Deep Neural Networks and Symbolic AI. *Nature* **2018**, *555*, 604–610.

(27) Steiner, S.; Wolf, J.; Glatzel, S.; Andreou, A.; Granda, J. M.; Keenan, G.; Hinkley, T.; Aragon-Camarasa, G.; Kitson, P. J.; Angelone, D.; Cronin, L. Organic Synthesis in a Modular Robotic System Driven by a Chemical Programming Language. *Science* **2019**, *363*, eaav2211.

(28) Mehr, S. H. M.; Craven, M.; Leonov, A. I.; Keenan, G.; Cronin, L. A Universal System for Digitization and Automatic Execution of the Chemical Synthesis Literature. *Science* **2020**, *370*, 101–108.

(29) Kusne, A. G. et al. On-the-Fly Closed-Loop Materials Discovery via Bayesian Active Learning. *Nat. Commun.* **2020**, *11*, 1–11.

(30) Burger, B.; Maffettone, P. M.; Gusev, V. V.; Aitchison, C. M.; Bai, Y.; Wang, X.; Li, X.; Alston, B. M.; Li, B.; Clowes, R.; Rankin, N.; Harris, B.; Sprick, R. S.; Cooper, A. I. A Mobile Robotic Chemist. *Nature* **2020**, *583*, 237–241.

(31) Hutter, F.; Hoos, H. H.; Leyton-Brown, K. Parallel Algorithm Configuration. International Conference on Learning and Intelligent Optimization. 2012; pp 55–70.

(32) Tran, K.; Ulissi, Z. W. Active Learning Across Intermetallics to Guide Discovery of Electrocatalysts for CO_2 Reduction and H_2 Evolution. *Nat. Catal.* **2018**, *1*, 696–703.

(33) Olson, R. S.; Urbanowicz, R. J.; Andrews, P. C.; Lavender, N. A.; Kidd, L. C.; Moore, J. H. Automating Biomedical Data Science Through Tree-Based Pipeline Op-

timization. European Conference on the Applications of Evolutionary Computation. 2016; pp 123–137.

(34) Christensen, M.; Yunker, L. P. E.; Adedeji, F.; Häse, F.; Roch, L. M.; Gensch, T.; dos Passos Gomes, G.; Zepel, T.; Sigman, M. S.; Aspuru-Guzik, A.; Hein, J. Data-Science Driven Autonomous Process Optimization. *Commun. Chem.* **2021**, *4*, 1–12.

(35) Gao, H.; Struble, T. J.; Coley, C. W.; Wang, Y.; Green, W. H.; Jensen, K. F. Using Machine Learning to Predict Suitable Conditions for Organic Reactions. *ACS Cent. Sci.* **2018**, *4*, 1465–1476.

(36) Rosen, A.; Iyer, S.; Ray, D.; Yao, Z.; Aspuru-Guzik, A.; Gagliardi, L.; Notestein, J.; Snurr, R. Q. Machine Learning the Quantum-Chemical Properties of Metal–Organic Frameworks for Accelerated Materials Discovery. *Matter* **2021**, *4*, 1578–1597.

(37) Taylor, C. J.; Booth, M.; Manson, J. A.; Willis, M. J.; Clemens, G.; Taylor, B. A.; Chamberlain, T. W.; Bourne, R. A. Rapid, Automated Determination of Reaction Models and Kinetic Parameters. *Chem. Eng. J.* **2021**, *413*, 127017.

(38) Waldron, C.; Pankajakshan, A.; Quaglio, M.; Cao, E.; Galvanin, F.; Gavriilidis, A. An Autonomous Microreactor Platform for the Rapid Identification of Kinetic Models. *React. Chem. Eng.* **2019**, *4*, 1623–1636.
